# Multiple genotypes of *Phelipanche ramosa* indicate repeated introduction to the Americas

**DOI:** 10.1002/ajb2.16456

**Published:** 2025-01-06

**Authors:** Adam C. Schneider

**Affiliations:** ^1^ Department of Biology University of Wisconsin, La Crosse La Crosse WI USA

**Keywords:** broomrape, ecotypes, genome skimming, host races, Orobanchaceae, parasitic plant, *Phelipanche nana*, *Phelipanche ramosa*, phylogeography, weeds

## Abstract

**Premise:**

*Phelipanche ramosa* is an economically damaging parasitic plant that has been reported in North America since the late 1800s. While this species comprises a variety of genetically distinct host races in its native range, the genetic composition of adventive populations in the New World remains unexplored. On the basis of morphological and ecological variation, some have suggested that the closely related *P. nana* may also be present.

**Methods:**

Genome skimming was used to assess the relationships of 30 populations of *Phelipanche* spanning the geographic and host ranges in North and South America, plus one *P. nana* reference population from Lebanon.

**Results:**

Phylogenetic analysis indicated four distinct genetic groups, though plastome and nrDNA data supported conflicting signals of relationships among them. First, specimens from Chilean tomato fields were nearly indistinguishable genetically from the reference *P. nana*. Second, a pair of samples from Virginia showed similar nrDNA as the first group, but divergent plastomes. The remaining 24 samples sorted into two groups, one which parasitizes cultivated plants, especially tomato, and the other on roadside weeds in different parts of the United States.

**Conclusions:**

The geographic and ecological cohesiveness of four distinct genetic groups supports a hypothesis of multiple introductions to the Americas, presumably from Eurasia, followed by little to no subsequent gene flow among them. However, such groups do not align with existing morphological or ecological species concepts for *P. ramosa* and *P. nana*. In practice, threat assessment of *Phelipanche* populations to agricultural settings should be evaluated regionally given the phylogeographic and ecological heterogeneity.

Among the broomrapes (*Orobanche* L. and *Phelipanche* Pomel; Orobanchaceae) that cause significant economic concern, *Phelipanche ramosa* (L.) Pomel (branched broomrape) parasitizes a wide variety of crops including tomato, hemp, carrot, celery, potato, lentil, rapeseed, tobacco, and sunflower. While native to Eurasia, in the last century, *P. ramosa* has spread to southern Africa, Australia, New Zealand, the United States, Cuba, and Chile (LaHalsted, 1901; Garman, 1903; Muenscher, 1924; Stout and Wagnon, 1953; Labrada, 1975; Musselman and Nixon, 1981; Matthei, 1995; Parker, 2009; Fernández‐Aparicio et al., 2016). Though severe countermeasures are typically taken to minimize spread in introduced areas, *P. ramosa* is nearly impossible to fully eradicate and can persist in seed banks for well over a decade (Stout and Wagnon, 1953; Habimana et al., 2014; USDA, 2019).

One factor contributing to its success in parasitizing a wide range of crop species is the presence of several genetically distinct but overlapping host races (Stojanova et al., 2019; Le Corre et al., 2023). In the Americas, *Phelipanche ramosa* primarily impacts cultivated tomatoes in California and Chile, tobacco in Cuba, and rarely hemp. At the same time, established populations of *Phelipanche* parasitize noncultivated forbs in fields and disturbed areas in parts of the southern and eastern United States (Figure 1; Musselman and Nixon, 1981; Musselman and Bolin, 2008; iNaturalist, 2024). The distinct ecology, as well as variation in certain morphological features, have led some to question whether such populations are instead the close relative *Phelipanche nana* (Noë ex Rchb.) Soják. These congeners are distinguished morphologically by floral traits such as the ratio of calyx tooth length to cup depth and by the amount of branching (Table 1), but intermediate specimens do exist (Foley, 2001b; Tzvelev, 2015). At present, *Phelipanche nana* is not recognized in the Flora of North America (Collins et al., 2016).

**Figure 1:**
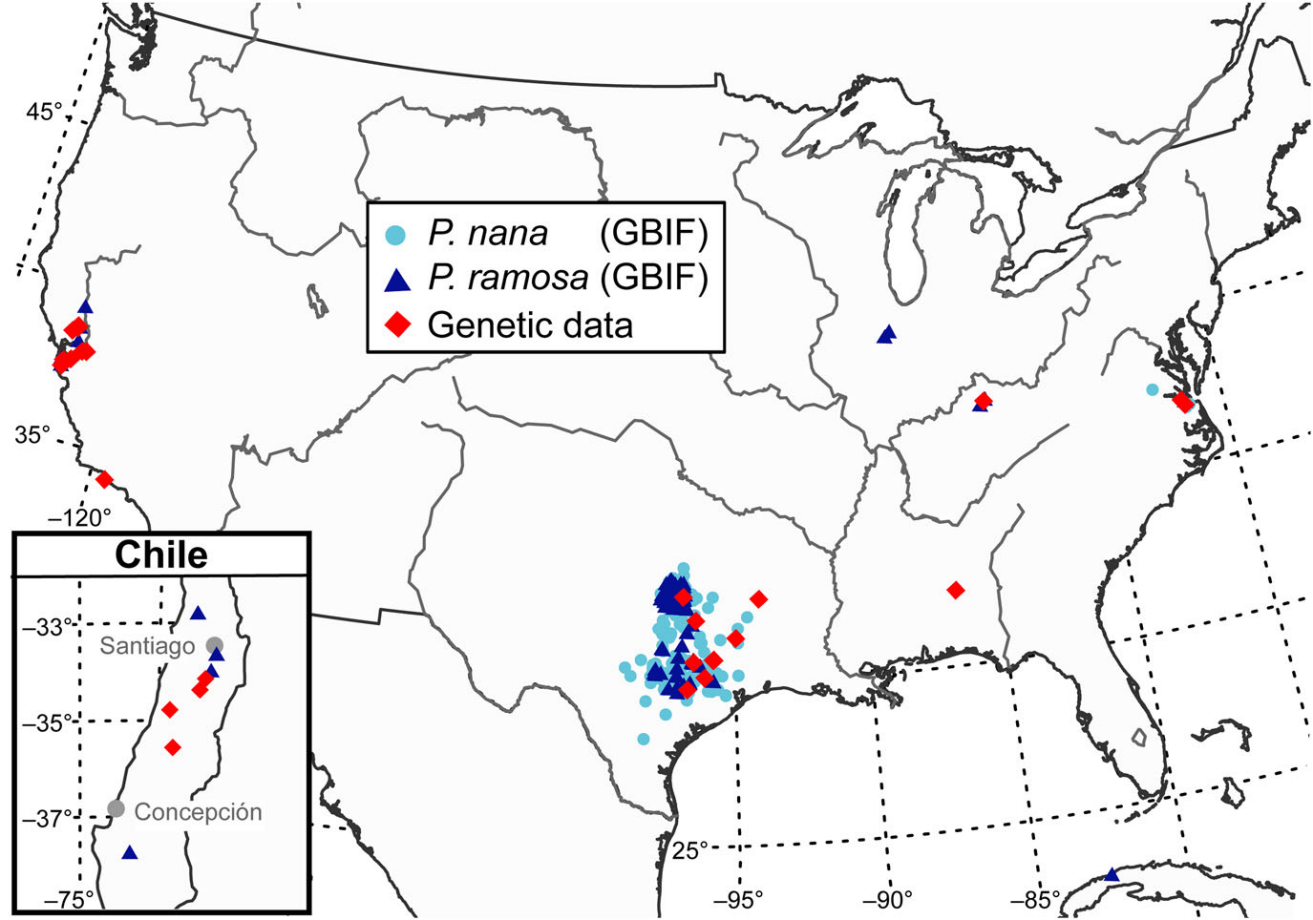
Georeferenced records of *Phelipanche* spp. in the United States, Cuba, and Chile (blue circles and triangles) with newly sequenced samples indicated by red diamonds. Data from GBIF.org (2024) with four iNaturalist and herbarium records from Chile manually added. Conflicting species determinations in the southern and eastern United States reflect controversy in identification rather than the presence of two co‐occurring species.

**Table 1:** Morphological and ecological comparison of *Phelipanche ramosa* and *P. nana* based on analysis of European specimens by Óscar Sánchez Pedraja.

Character	*P. ramosa*	*P. nana*
Stem	20–35(–40) cm	15–20(–30) cm
	Very branched from the base; rarely with bulbous base	Simple or rarely branched; frequently with bulbous base
Inflorescence	>10 cm long	<8 cm long
	Equal to or longer than the rest of the stem; flowers lax	Equal to or shorter than the rest of the stem; flowers dense
Flower presentation	Erect	Erect‐patent
Calyx teeth	Equal in length to the calyx tube; tips acute	Longer than the calyx tube; tips acuminate
Corolla: dorsal line	+/– Straight or slightly curved	Clearly gibbous
Corolla lobes	Ovate with rounded tips	Ovate with acute tips
Corolla color	Pale (blue to violet)	Deep blue; more rarely pale
Host/habitat	Cultivated crops	Wild plants in meadows or ruderal areas
Representative specimens: herbarium barcode	CLF170347 P03996068 P04384578 US03926537 P03996068 AMD84207	WU0035396 (syntype) K000806971 E00023129 BRIT713678

Despite the recent dispersal history and present threat to agricultural systems, relationships among populations of introduced *Phelipanche* in the Americas have not been investigated genetically. In Europe by contrast, a variety of genetic tools have been used to distinguish *P. ramosa* from close relatives like *P. nana*. These include RAPD (randomly amplified polymorphic DNA; Román et al., 2003) and to a lesser extent, phylogenetic analyses with the ITS and *trnL‐trnF* spacer in the plastid genome (Carlón et al., 2008; Piwowarczyk et al., 2021). In this study, I used a genome‐skimming approach to compare the complete plastid genome and nuclear ribosomal tandem repeat (including ITS) of 30 samples previously identified as *P. ramosa* across the United States with reference sequences of European *Phelipanche ramosa*, *P. nana*, and close relatives.

## MATERIALS AND METHODS

Thirty‐one specimens of *Phelipanche* were selected for genomic analysis (Table 2). Thirty of these specimens cover the known geographic range in the United States and Chile (Figure 1) and the reported variation in host associations (e.g., *Solanum* spp*., Cannabis sativa* L., non‐crop forbs), date of collection (1929–2023), and morphology (most notably the degree of inflorescence branching from unbranched to highly branched and floral color from pale to deep purple). The remaining specimen sequenced was a reference collection of *P. nana* collected in Lebanon. No sample from Cuba (parasitizing tobacco) could be obtained, and an earlier report of *P. ramosa* in Mexico (Musselman, 1980) could not be verified with a specimen or primary source reference. On the contrary, specimens of *Aphyllon cooperi* parasitizing tobacco near Nayarit, Mexico had been misidentified as *P. ramosa* and other taxa, which I determined from morphology and DNA sequencing (GenBank PQ496880).

**Table 2:** Voucher information and hosts for 30 *Phelipanche ramosa* samples and one outgroup of *P. nana*. Host/ecology information derived from herbarium specimens or personal communication with collectors. Herbarium abbreviations follow Index Herbariorum, with barcode or accession number if available.

Genetic Group	Collector (Locality)	Herbarium	Host/Ecology	GenBank accessions	
				nr	pt
*Phelipanche nana*	Howard C. Stutz 3199 (Lebanon)	ISC 275006 (dup. at BRY)	Streamside; host unknown	PQ479131	PQ611166
Group A	Juan Carlos Galaz s.n. (Chile: Cachapoal: Rancagua)	no voucher	On tomato	PQ479130	PQ611170
	Juan Carlos Galaz s.n. (Chile: Cachapoal: Quinta de Tilcoco)	no voucher	On tomato	PQ479133	PQ611171
	Juan Carlos Galaz s.n. (Chile: Colchagua: Lolol)	no voucher	On tomato	PQ479132	PQ611172
	Juan Carlos Galaz s.n. (Chile: Talca)	no voucher	On tomato	PQ479134	PQ611173
Group B	Zach Bagley (California)	no voucher	On tomato	PQ479113	PQ611155
	W. M. Shrader (California)	CDA 0006504	On *Solanum physafolia* (*S. sarachoides* misappl.)	PQ479114	PQ611185
	A. Moretto (California)	CDA 0006504	On tomato	PQ479115	PQ611165
	C. O. Howe (California)	CDA 0006509	On tomato	PQ479116	PQ611161
	D. B. Buettner (California)	CDA 0006502	On tomato	PQ479117	PQ611158
	W. B. Kirk (California)	DAV 351557	On tomato	PQ479118	PQ611184
	Robert Pelletier (California)	CDA 0042361	On tomato	PQ479119	PQ611168
	Gene Miyao (California)	DAV 219788	On tomato	PQ479120	PQ611164
	Schneider 1332 (California)	DAV 244972	On tomato	PQ479121	PQ611182
	Schneider 1333 (California)	DAV 244973	On tomato and *Solanum physafolia*	PQ479122	PQ611183
	Brad Hanson (California)	*no voucher*	On tomato	PQ479123	PQ611160
	F. T. McFarland s.n. (Kentucky)	BRIT 991519	On *Cannabis sativa*	PQ479128	PQ611163
Group C	Patrick Baldwin 9184 (Virginia)	WILLI 83158	Lawn area next to drainage ditch; host unknown	PQ479135	PQ611167
	J. Bolin et al. 07‐20 (Virginia)	ODU 14286 (dups. at NCU, VPI)	On *Medicago lupulina*, located in a mowed field	PQ479136	PQ611156
Group D	Pickens 28 (Texas)	BRIT 991491	Roadside; host unknown	PQ479127	PQ611169
	Randle 222 (Texas)	SHST	Randle populations on *Sherardia arvensis, Medicago lupulina, Melilotis officinalis* (uncommon), *Verbena halei* (rare)	PQ479107	PQ611175
	Randle 223 (Texas)	SHST		PQ479108	PQ611176
	Randle 224 (Texas)	SHST		PQ479109	PQ611177
	Randle 225 (Texas)	SHST		PQ479110	PQ611178
	Randle 226 (Texas)	SHST		PQ479111	PQ611179
	Brown 31973 (Texas)	BRIT 991495	Disturbed places near road with *Medicago* sp., and *Sherarida arvensis*; host unknown	PQ479124	PQ611157
	Rosen 3316 (Texas)	BRIT 991489	Roadside; host unknown	PQ479126	PQ611180
	Ruhlman and Jansen RJ376 (Texas)	DAV (dup. at TEX)	Open meadow; host unknown.	PQ479106	PQ611181
	Quayle 529 (Texas)	BRIT 991493	Roadside; host unknown	PQ479125	PQ611174
	Diamond 27259 (Alabama)	BRIT 558675 (dup. at TROY)	Grassy roadside; host unknown	PQ479129	PQ611159
	J. M. Kelley s.n. (Louisiana)	LSU 00219949	Associated with ruderal, native herbs and European exotics; host unknown.	PQ479112	PQ611162

Previously extracted DNA from five *Phelipanche* collections in Texas was sent by Chris Randle (Sam Houston State University, Texas, USA). From the remaining 26 specimens, dried flowers or an inflorescence tip from single individuals were ground using steel beads and a Beadbug 6 homogenizer (Benchmark Scientific, Sayreville, NJ, USA). DNA was then extracted using a DNeasy Plant Pro kit (Qiagen, Valencia, CA, USA) following the manufacturer's instructions. Aliquots of DNA were then sent to the University of Wisconsin Biotechnology Center DNA Sequencing Facility for library preparation and 2 × 150 paired‐end sequencing using approximately 3/8 of a Novaseq X Plus lane (Illumina, San Diego, CA, USA) or to Novogene (Sacramento, CA, USA) for library preparation and Novaseq X Plus sequencing at a similar coverage depth. For each sample, 57.2 million paired reads were generated (mean; range 24–97 million) and deposited in the NCBI sequence read archive (BioProject PRJNA1176015; experiments SRX26455412–SRX26455442).

From each set of reads, the nuclear ribosomal repeat and plastome were assembled de novo using GetOrganelle v.1.7.4.1 (Jin et al., 2020) run on Python v.3.8.2 with dependencies Bowtie2 v.2.4.1, SPAdes v.3.12.0, and Blast v.2.6.0. When multiple contigs were returned instead of one complete sequence, these were joined by comparison with congeneric reference sequences. Alternative orientations of a 15‐kb fragment from *rps7* to *rps15* flanked by a 115‐bp inverted repeat were manually adjusted to a standard orientation. nrDNA and plastome alignments were performed using MAFFT v.7.505 on XSEDE via the CIPRES Science Gateway (Katoh and Toh, 2010; Miller et al., 2010), then manually inspected.

Phylogenetic analysis of the respective nrDNA and plastome alignments was then performed using RAXML‐NG v.1.2.2 (Kozlov et al., 2019)*. Phelipanche aegyptiaca* (Pers.) Pomel was used as an outgroup for the plastome phylogeny, and an additional sample of *P. ramosa* that was cultivated on a tomato at the Botanical Garden in Bonn, Germany was added as a reference sequence (GenBank #NC_023465; voucher BONN: S Wicke Pr52/53; Wicke et al., 2013). The nrDNA tree was rooted based on a genus‐level analysis of the ITS locus, detailed below. Statistical support for each clade was assessed using 1000 bootstrap replicates.

Neither plastome nor complete nrDNA repeat sequences are available for most species of *Phelipanche*. Therefore, new samples could only be compared to previously sequenced populations at the ITS region of nrDNA and the *trnL‐trnF* spacer region of the plastome. Acquired sequences came from three sources: curated alignments published by Piwowarczyk et al. (2021), the ITS from 22 individuals in cultivated tomato fields in California (GenBank OR690545–OR690569; voucher specimens at CDA), and the ITS from six specimens parasitizing tobacco in Bulgaria (GenBank MK024283–MK024290; Kirilova et al., 2019). Phylogenetics trees were generated using RAxML‐NG as above and rooted following Piwowarczyk et al. (2021).

## RESULTS

**Figure 2:**
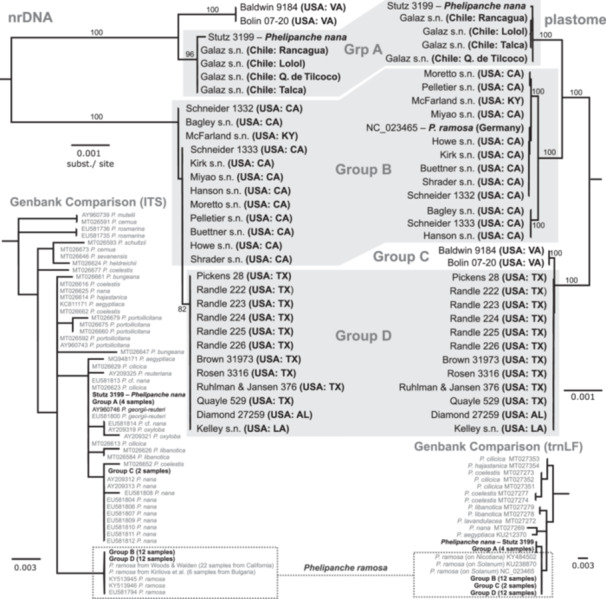
Nuclear ribosomal repeat (nrDNA) and plastome phylogenies of 31 samples of *Phelipanche ramosa* from the United States and Chile and a reference sample of *P. nana* from Lebanon. Tip labels indicate collector and locality, with voucher information available in Table 2. Bootstrap support of selected nodes shown. Inset: Phylograms comparing samples shown in the main nrDNA and plastome panels (black tip labels) with a curated selection of previously published *Phelipanche* spp. (gray labels showing GenBank accession and taxon). Rooting follows Piwowarczyk et al. (2021). AL, Alabama; CA, California; LA, Louisiana; TX, Texas; VA, Virginia.

Phylogenetic analysis revealed four genetic groups that were strongly aligned with geography and sometimes discordant to species boundaries as determined by morphology (Figures 1, 2). Group A comprised Chilean specimens that parasitize tomatoes from four distinct locations spanning ~175 km and have nearly identical nrDNA and plastome sequences to the reference *Phelipanche nana* from Lebanon.

Group B comprised Californian specimens parasitizing *Solanum* spp. and the specimen on *Cannabis sativa*. These 12 samples formed a strongly supported clade in the plastome analysis along with a reference sequence of *P. ramosa* from Germany (bootstrap = 100%) and had nearly identical nrDNA haplotypes, differing only by a single base substitution.

Group C comprised two specimens from the Chesapeake Bay region of Virginia that have more complex genetic affinities. The ITS regions of these specimens were identical to those of several *P. nana* specimens from Mediterranean countries and more closely related to Group A than to any other specimens sampled (bootstrap = 100%, Figure 2). However, the plastomes of Group C were nearly identical to those of Group D, which is composed of additional specimens in the south central and southeastern United States, including Texas.

While nrDNA relationships suggest that the Chilean populations and Group C are genetically *Phelipanche nana*, plastome relationships more closely paralleled ecological differences among the samples: Groups A and B mostly parasitize cultivated tomato (but see Stout and Wagnon, 1953), while Groups C and D are naturalized on a wider variety of ruderal hosts (Musselman and Nixon, 1981).

## DISCUSSION

### Phylogeography of American *Phelipanche*

The consilience of four genetic groupings of *Phelipanche* (Figure 2) with their ecological and geographical context within North and South America (Table 2) supports a hypothesis of multiple introductions. Presumably, the source of introduction in each of the cases was from Eurasian progenitors; otherwise, one would expect higher‐level phylogenetic structure among exclusively American populations supported by both nrDNA and plastome data sets. The clear geographical differentiation of genetic groups also illustrates the limited natural dispersibility among populations, consistent with other studies of *P. ramosa* (Román et al., 2003; Stojanova et al., 2019).

In Europe, *Phelipanche ramosa* is composed of several genetically distinct races, each with distinct host profiles (Stojanova et al., 2019; Le Corre et al., 2023). Similarly, in North America, parasites in genetic Group B primarily parasitize cultivated processing tomatoes and sometimes the co‐occurring weed *Solanum physalifolium* Rusby. Historically, other species may have been hosts of Group B as well (Stout and Wagnon, 1953). Group A plants also parasitize cultivated tomatoes in South America. By contrast, Group C and Group D specimens parasitize a variety of non‐cultivated forbs in fields or ruderal areas, particularly *Medicago* L. Some contemporary or historical populations cannot be placed into a genetic group. No specimens parasitizing cultivated tobacco from eastern North America or Cuba could be obtained. Additionally, early infestations of *P. ramosa* were reported on coleus and tomatoes in the northeastern United States, but these populations were completely eradicated before voucher specimens could be made (Halsted, 1901; Garman, 1903; Muenscher, 1924).

Although the various races identified within European *P. ramosa* had overlapping host preferences, one cluster was strongly associated with hemp (cluster 4 of Le Corre et al. [2023] and cluster 2a of Stojanova et al. [2019]), and all tomatoes were associated with a single cluster that also included tobacco (cluster 5 of Le Corre et al.) or tobacco and other hosts (2b of Stojanova et al.). By contrast, a single genotype of *P. ramosa* appears responsible for outbreaks in the United States on tomato and at least some hemp crops dating back to the early 20^th^ century (Group B, Figure 2). However, it is highly distinct from the lineage that has been parasitizing tomatoes in Chile since the 1980s (Group A; Matthei, 1995). Lack of comparable genetic markers currently limit the ability to further compare American specimens with these previously described European races, but should be prioritized in future research. In any case, the strongly supported plastid, and to a lesser extent nrDNA clades shown here (Figure 2), coupled with lack of intermediate genotypes at neutral loci solidly indicates a lack of gene flow between these four genetic groups in the Americas.

### Taxonomic implications for the *P*. *ramosa* complex

Relationships among the four genetic groups present in the Americas do not clearly align with existing morphological or ecological species concepts for *P. ramosa* or *P. nana* (Figure 2; Sánchez Pedraja et al., 2016). The close relationship of these two taxa is indisputable: *Phelpanche nana* was first recognized as *P. ramosa* var*. simplex* F.W. de Noë ex Vis., based on morphological characters and its parasitism of *Trifolium scabrum* (Sánchez Pedraja et al., 2016). Recently, *P. nana* has also been treated as a subspecies of *P. ramosa* along with the more distinct *P. mutelii* (F.W. Schultz) Pomel.

While *P. mutelii* is more distantly related to the other two species (Román et al., 2003), *P. ramosa* may be a recently adapted form of *P. nana* that specializes on cultivated plants (Tzvelev, 2015). Early descriptions of *P. ramosa* indicate its parasitism of hemp crops (“observanimus…in agris Cannabi”; Chabrey, 1677, p. 257), with later references encompassing other cultivated plants including tobacco (Reichenbach, 1829), tomato (Beck, 1890), and several others (Le Corre et al., 2023). Such recent incipient and adaptive speciation of *P. ramosa* could be consistent with the patterns of introgression, lack of reciprocal monophyly, and conflict between morphology and genetics indicated by this study (Figure 2).

Nevertheless, the complex genetic relationships of American *Phelipanche* (Figure 2) strain the utility of the morphological or ecological species concepts currently used to distinguish *P. ramosa* and *P. nana*. While claims of *P. nana* in North America had previously been based in part on the host ecology of Group C and D populations, Chilean populations on cultivated tomato (Group A) are nearly genetically identical to the *P. nana* reference sample from Lebanon. Environmental conditions in a cultivated field, including more vigorous hosts and shading could contribute to the more “ramosa‐like” robust, branched plants and paler flowers that are often observed in those areas. However, plants within a single population can range in floral color from pale to dark blue (J. C. Galaz, UC Davis Chile Life Sciences Innovation Center, personal communication), but although smaller individuals of a variety of *Phelipanche* species can be easily misidentified as *P. nana* (Foley, 2001a), morphological traits correlate well with distinct host ecologies (Table 1). A common garden experiment could test the extent to which these traditional taxonomic characters have a genetic basis.

While these four genetic groups do not appear to co‐occur spatially within the Americas, conflict between plastome and nuclear ribosomal data (Figure 2) indicates possible introgression between populations parasitizing cultivated and uncultivated hosts preceding dispersal into the Americas. Recent post‐dispersal hybrids would be expected to retain identical plastome haplotypes to their maternal parent, but both Groups C and D have distinct terminal branches that indicate genetically fixed differences compared to American relatives. This discordance among adventive populations illustrates the need for a global multilocus genetic analysis of these taxa, along with their close relatives across their extensive geographical and host ranges to clarify lineage boundaries (or the lack thereof) before assessing diagnostic morphological differences to support improved taxonomic keys and descriptions.

### Complex plastome structure

Although analyzing genomic content or structure was not the primary purpose of this study, the *P. ramosa* plastomes sequenced here notably could not be assembled as circular. Based on their GetOrganelle assembly graph, they may have more complex structure than the canonical quadripartite plastome. Wicke et al. (2013) reported the loss of the large inverted repeat in their representative sample of this species. In our analysis, the longest contig of each sample, which ranged from 32,233 and 53,753 bp, had a coverage twice that of others. Unlike the typical boundaries of the inverted repeat, this region comprises some or most of the genes found in the large single copy region of most plastomes and, in some cases, extends into the rDNA portion of the inverted repeat. Additionally, a 115‐bp inverted repeat flanking a 15‐kb fragment from *rps7* to *rps15* led to alternative assembly conformations in some samples.

### Management implications

*Phelipanche ramosa* is considered one of the worst parasitic weeds of agricultural crops and is on many state and national noxious weed lists (USDA, 2019). By contrast, *P. nana* is generally considered of more modest economic concern despite its occasional presence on crop species (Schneeweiss, 2006; Vagelas and Gravinis, 2014; Crescenzi et al., 2015). Differences in the host range and ecology of the four genetic groups in the Americas suggest that each has a differential capacity for spreading to new hosts or areas. Groups B and C are largely generalists, parasitizing many native and naturalized forbs in a single population (Table 2; Musselman and Nixon, 1981). On the other hand, Group B only have been observed parasitizing *Solanum* and cultivated hemp, even though many of the weedy Group D hosts are also present in California and vice versa.

Despite evidence of multiple introductions to the Americas, genetic and geographic uniformity within each group indicates that gene flow is not occurring at continental scales. Therefore, agricultural risk assessments may be most effective if done on a regional basis that accounts for the unique ecological and host differences in different areas. For example, the historical record of genetic Group B parasitizing hemp in Kentucky (McFarland s.n.) indicates that *P. ramosa* in Californian tomato fields may be a potential threat to the growing cannabis industry in California, given the increasing abundance of cultivation sites in California and their proximity to infected tomato fields (Department of Cannabis Control, 2023).

## AUTHOR CONTRIBUTIONS

**Adam C. Schneider**: Conceptualization; Data curation; Formal analysis; Funding acquisition; Investigation; Methodology; Project administration; Visualization; Writing.

## Data Availability

Herbarium vouchers and GenBank accession numbers are cited in Table 2. Additional specimen data, sequence alignments, and the maximum likelihood tree files used to make Figure 2 are available in the Dryad Digital Repository: https://doi.org/10.5061/dryad.cvdncjtdg. Raw Illumina reads can be downloaded from the NCBI Sequence Read Archive BioProject PRJNA1176015 (experiments SRX26455412–SRX26455442). A preprint version of this article is available on Zenodo under a CC‐BY‐NC license: https://doi.org/10.5281/zenodo.14556553.
